# Rhenium/rhenium oxide nanoparticles production using femtosecond pulsed laser ablation in liquid

**DOI:** 10.3906/kim-2008-59

**Published:** 2021-04-28

**Authors:** Abdullah KEPCEOĞLU, Yasemin GÜNDOĞDU, Adem SARILMAZ, Mustafa ERSÖZ, Faruk ÖZEL, Hamdi Şükür KILIÇ

**Affiliations:** 1 Department of Physics, Faculty of Science, Selçuk University, Konya Turkey; 2 Department of Computer Technologies, Kadınhanı Faik İçil Vocational High School, University of Selçuk, Konya Turkey; 3 Department of Metallurgical and Materials Engineering, Faculty of Engineering, Karamanoğlu Mehmetbey University, Karaman Turkey; 4 Directorate of High Technology Research and Application Center, Selçuk University, Konya Turkey; 5 Department of Chemistry, Faculty of Science, Selçuk University, Konya Turkey; 6 Selçuk University Laser Driven Proton Therapy Research and Application (SULTAN) Center, Konya Turkey

**Keywords:** Rhenium nanoparticles, ReO_3_, oxide nanoparticles, laser processing, pulsed laser ablation

## Abstract

In this study, rhenium/rhenium oxide nanoparticles (Re / ReO_3_ NPs) have been produced for the first time in ultrapure water by using Femtosecond Pulsed Laser Ablation in Liquid (fsPLAL) method. X-Ray Diffraction (XRD) measurements and results obtained for NPs show the existence of well-crystallized peaks and preferred phases. Re NPs have hexagonal structure while ReO_3_ NPs have the perovskite-like cubic crystal structures. The Re / ReO_3_ ratio is also determined to be 53 / 47 with ~ 20 nm crystallite size, while pure ReO_3_ crystallite sizes were measured to be ~ 25 nm. The TEM results have shown that the produced particles have a spherical shape, and particle sizes changes between ~ 20 nm and ~ 60 nm. The crystallite size is similar due to XRD results. Obtained nanoparticles exhibit promising applications for photonic devices with broad bandgap values which have measured to be 4.71 eV for Re / ReO_3_ NPs mixture and 4.36 eV for pure ReO_3_ NPs.

## 1. Introduction

Pulsed laser ablation (PLA) of solids is a very promising functional, powerful, and clean method for nanoparticle production [1]. NPs with controlled sizes, shapes, and concentrations can be obtained using PLA. PLA in liquid (PLAL) method becomes a facile and clean (free of contaminats) method to produce NPs [2] because this method can easily be used to produce NPs in a reservoir of ultrapure water, water surfactant mixtures or some other liquid materials [3]. Metallic NPs may have very broad area of applications as such copper [4] and silver [5] could be used as an antibacterial potency against *E. coli*. Especially, gold NPs were extensively used in very broad application area including catalysis, nanotechnology [6], cancer diagnostics [7], and biological applications [8]. Also, Au NPs are widely used in thin film applications [9,10] and in nonvolatile memory devices [11]. 

In recent years, rhenium nanoparticles (NPs) have been used in several applications such as a tumour treating therapies and coating (plastics, metals, textiles) technologies as well as magnetic rhenium NPs, which have been used as a contrast agent and are produced by using chemical or physical methods. Rhenium containing NPs have been studied and reported for catalytic and sensor applications in literature [12,13]; platinum monolayer on iridium/rhenium alloy nanoparticles [14] may function as a core part of some core-shell structures for the oxygen reaction, rhenium-containing Polytetrafluoroethylene (PTFE) structures [15] as catalysts and ReO_3_ @ SiO2, ReO_3_ @ Ag, ReO_3_ @ Au NPs for sensor applications [16]. There are a few methods reported in the literature for production of Re NPs, which are pulsed laser decomposition [17], reduction of some organometallic complexes [18] or colloidal and microemulsion synthesis in liquid environment [19], but, due to the author’s knowledge, no study has yet been reported in the literature about the use of the fsPLAL method for production of Re NPs as well as ReO_3_. Rhenium-containing alloys and coatings have been used for high-temperature applications, while Re NPs have also been used to connect parts at lower temperatures [20], which reduces the melting point of alloys. In a recent study, amorphous RexOy NPs were produced in tunable particle sizes by using gamma radiation [21]. ReS NPs were synthesized as magnetic nanoparticles [22,23] for the contrast agent. In comparison with other transition metal oxides, ReO_3_ has some attractive properties such as having a perovskite-like cubic structure and higher conductivity than metallic rhenium [24]. The resistivity of the single-crystal bulk ReO_3_ is about (8.95 ± 0.03) × 10–6 Ω∙cm at 300 K [25]. The optical properties of ReO_3_ Nano-Crystals (NCs) were studied by Biswas and Rao [26]. The colloidal ReO_3_ NCs exhibits very high enhanced localized surface plasmon resonance (LSPR) in the visible range of spectrum from 488 nm to 534 nm that is comparable to gold NPs LSPR range, and ReO_3_ NPs show some metallic behaviours [27]. In a recent study, 8% ReO_3_ NPs doped in 1,1-bis-(4-bis(4-methyl-phenyl)-amino-phenyl)-cyclohexane (TAPC) were used as a p-doped hole injection layer (HIL) in transparent organic light emitting diodes (OLED) [28]. Surface plasmon resonance (SPR) nature of ReO_3_ NPs has been reported; it has been shown that SPR peaks of NPs lies in the visible range, and it can be tuned over the relevant spectral range by controlling the size of NPs. When particle size tuned from 8.5 nm to 32 nm, SPR peak is also tuned from 490 nm to 540 nm [26]. Recently, researchers are very interested in the production of complex NPs from bulk materials using facile and single step production methods that allow choosing parameters. In this study, the single step facile production of the transition metal/metal oxide (Re / ReO_3_) NPs was performed by using fsPLAL method and characterizations together with the interpretation of NPs obtained.

## 2. Materials and methods

In this work, we have used a Ti:Sapphire femtosecond laser system (Quantronix, Integra-C-3.5, NY, USA) pumped by Kerr-Lens mode-locked Ti:Sapphire laser (Quantronix, Ti-Light, NY, USA) (with 330 mW pulse power). Parameters are given as follow: amplifier laser delivers mode-locked chirped laser pulses with up to 3.5 W per pulse at a wavelength of 810 nm and with 90 fs pulse duration, and 1-3 kHz repetition rate with 8 mm beam diameter. Detailed system parameters were described elsewhere [29].

The laser output was adjusted and controlled by using an oscilloscope (WaveRunner 64 Xi, four channel digital-storage oscilloscope, LeCroy Corporation, NY, USA) triggered with a fast photodiode (Alphalas, UPD‐35‐UVIR‐D, Germany). 0.125 mm thick 99.99% pure rhenium (Goodfellow) target translated to the focal point by using a motorized lab jack (MLJ050, ThorLabs, Newton NJ, USA). We have used micromachining system to scan rhenium target and set laser pulse power to 800mW. Re NPs were produced in ultrapure deionized water (18.3 MΩ∙cm) (Millipore, USA) with 1 cm thick water layer above sample (8.04 cm3). 

**Figure 1:**
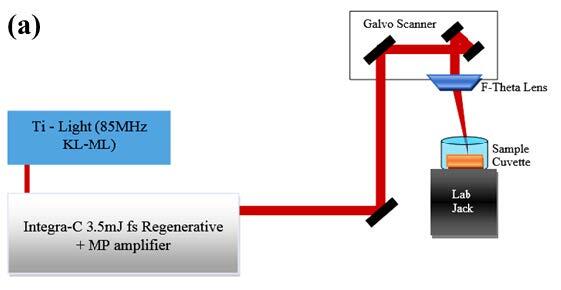

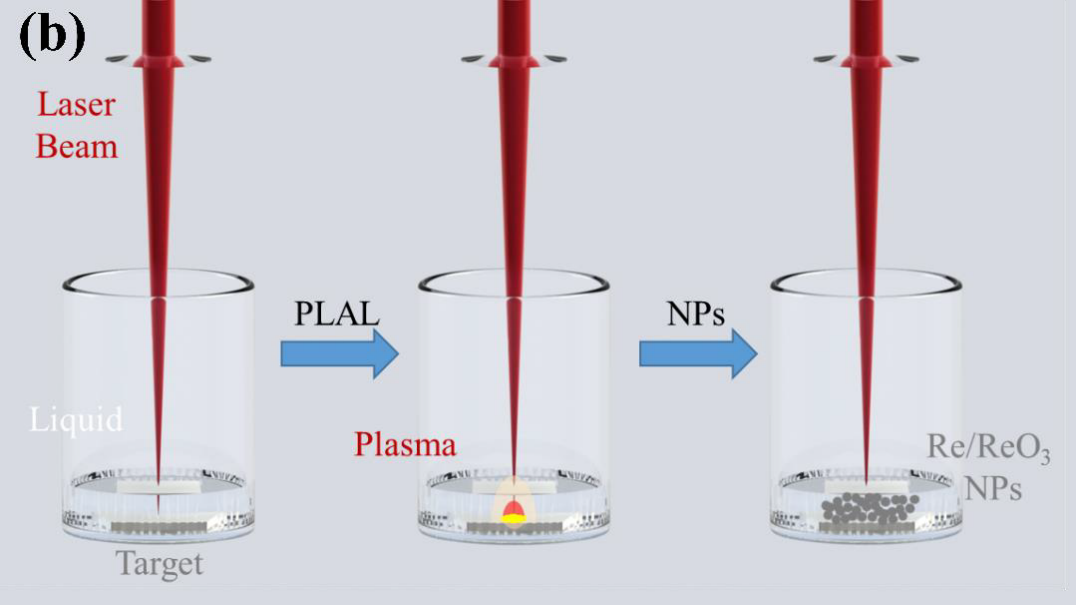
Femtosecond PLAL (a) nanoparticle production setup, (b) NP production using PLAL method.

The experimental setup is shown in Figure 1(a) and PLAL method briefly introduced as depicted in Figure 1(b). In PLAL method, NPs were produced by courtesy of PLAL method from Re plate sinked in liquid applying pulsed fs laser beams. Target located above the target holder when the laser beam enters the water vertically. If laser beam comes horizontal direction, one can use rotating cylindrical or fixed flat surfaced targets. In this work, vertical laser beams were used to ablate target material inside ultrapure water. As a usual case of interaction between fs laser pulses and target, resonance laser ablation (RLA) process is dominated by photoionization processes (single or multiphoton processes) and following locally induced space-charge separation fields and electron-ion collisions cause the formation of cavitation bubble [30]. There is two step in PLAL method, first one is plasma production and the last one is the continued growth of the NPs after collapsing of the plasma [31].

Laser-irradiated 10 × 10 mm2 surface area of target was put inside the container and 10 mm height ultrapure water added. Marking systems laser input was power reduced to 800 mW, pulses were used at 1 kHz repetition rate and wavelength was 800 nm. Scanning parameters were set to 3 mm∙s–1 for scanning speed, 250 μm line gap between each scan, working area set to 6 × 6 mm2 and each line scanned twice.

## 3. Results and discussion

**Figure 2:**
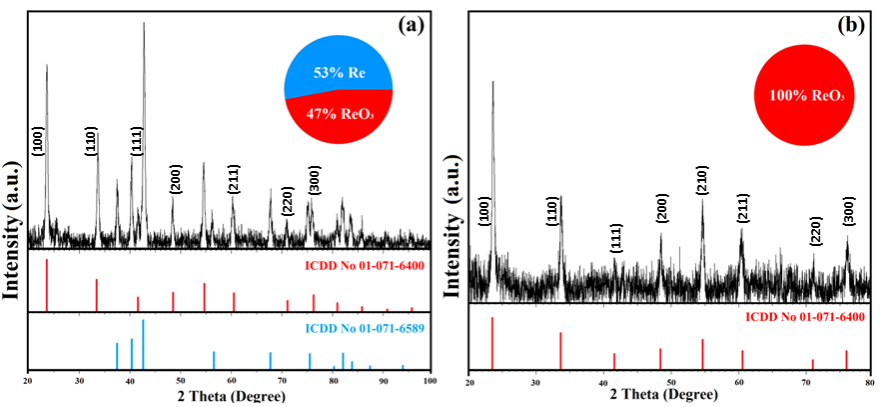
XRD pattern of (a) the Re / ReO3 NPs produced in 30 min, (b) ReO3 NPs were produced in 60 min with low water volume.

The crystal structures of Re / ReO_3_ nanoparticles produced in this study have been examined using XRD technique (Bruker AXS, Bruker D8 Advance, Germany), and XRD results of powdered NPs are shown in Figure 2.

Figure 2(a) shows XRD spectrum for Re / ReO_3_ NPs mixture in a ratio of 53 / 47 produced within 30 min. Diffraction peaks of the produced nanocrystals are intense and neat, showing that the Re NPs have hexagonal (space group: P63 / mmc) structure while ReO_3_ NPs have the perovskite-like cubic (space group: Pm-3m) structure [32] with effective crystallization and lack of impurities. The crystallite size of the obtained NPs measured from XRD spectra was calculated using Debye–Scherrer equation, size = Kλ/β cosθ, where, K is a dimensionless constant, β is full width at half maximum (FWHM in radian), θ is the diffraction Bragg angle and λ is the wavelength of X-ray in equation [33]. The average crystallite size was calculated due the most intense peaks to be 21 nm for ReO_3_ and 22 nm for Re using the Debye-Scherrer equation.

XRD results of the ReO_3_ NPs are shown in Figure 2(b). The diffraction peaks at 2θ = 23.7˚ (100), 33.7˚ (110), 41.7˚ (111), 48.5˚ (200), 54.7˚ (210), 60.4˚ (211), 71˚ (220) and 76˚ (300) are consistent with the standard peaks and index for ReO_3_ in literature [34]. All of the samples show semi-broad diffraction peaks and these broad peaks are due to the small size of the ReO_3_ nanocrystals [35]. The average crystallite size of ReO_3_ NPs was found to be about 25 nm.

**Figure 3:**
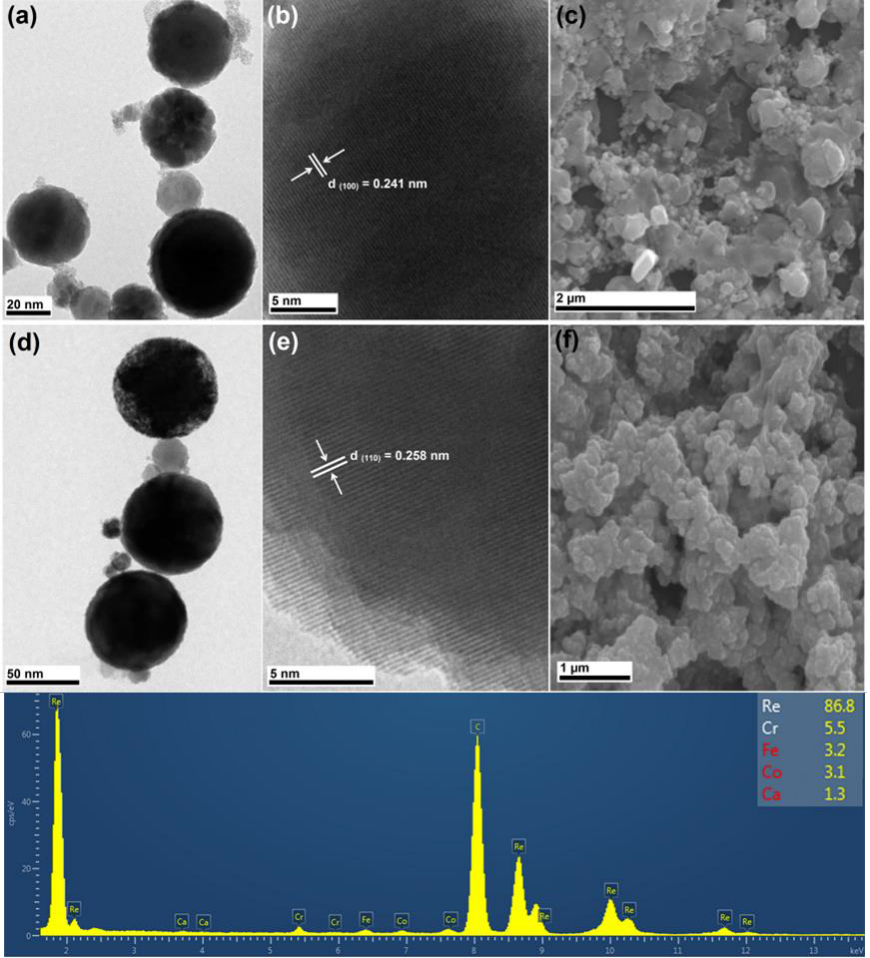
Re / ReO3 NPs (a) TEM, (b) HR-TEM, (c) SEM images and pure ReO3 NPs, (d) TEM, (e) HR-TEM, (f) SEM images, and (g) EDX Spectra of the NPs show ~86.8% Re, and other metals (Fe, Cr, Co, Ca impurities of the bulk sample) for NP are shown.

TEM (JEOL JEM-2100F, Japan) and SEM (Zeiss LS 10 - Carl Zeiss NTS GmbH, Germany) analyses have been carried out to present some further properties of the NPs as seen in Figure 3. TEM results of Re / ReO_3_ and ReO_3_ NPs are given in Figure 3(a) and (d), respectively. According to these results, produced particles have a spherical shape and particle sizes changes from ~ 20 nm (similar to that obtained from XRD crystallite sizes) to ~ 60 nm. Furthermore, HR-TEM analyses were carried out to investigate the crystal structure of Re / ReO_3_ and ReO_3_ NPs, and these results are given in Figure 3(b) and 3(e). As can be clearly seen from Figure 3(b) and 3(e), atoms constituting the lattice fringes are perfectly arranged and it shows that NPs have highly crystalline nature. The inter-planar spacing of Re and ReO_3_ NPs were measured as 5.41 and 2.58 Å, respectively. It is seen that these are due to (100) and (110) crystallographic planes, respectively.

Moreover, samples prepared for SEM imaging were first centrifuged at 1200 rpm for 10 min, NPs were sprinkled to double-sided adhesive band stick on the sample stub and then blow away the loose excessive particles and then samples were coated with gold using sputter coater. SEM images of NPs given in Figure 2(c) and 2(f) show that an increase in duration of the NP production causes solvent evaporation during the sample preparation process and then an increase in the aggregation of NPs. A condensation of the solution after evaporation of the liquid by heating may cause the highly aggregated nanoparticles. Furthermore, as can be seen clearly from Figure 3(c) and 3(f), pure ReO_3_ have a homogeneous structure than Re / ReO_3_ mixture.

An increase in the sample concentration can cause some increase in the number of photons absorbed by the NPs. This difference in the concentration can also result in an increase in the refractive index of the medium and stronger nonlinear effects like white light continuum (WLC) can be generated [36], and then this may cause some fragmentation of the particles to smaller sized fragments, and therefore, suppresses the aggregation [37]. In this study, in addition to WLC generation, some agglomeration after the end of irradiation was observed dramatically increased in time.

**Figure 4:**
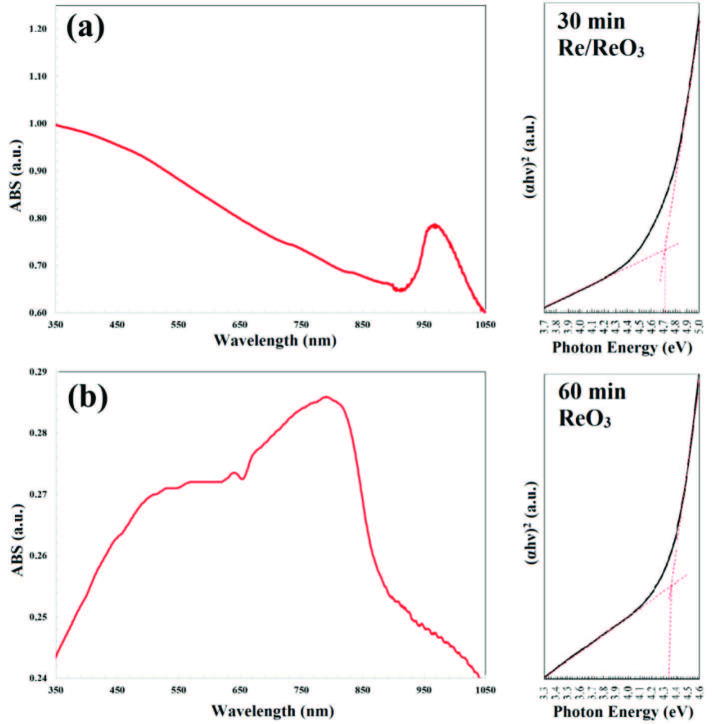
UV-Vis absorption spectra and Tauc-Plots; (a) Re / ReO3 NPs ablated for 30 min., (b) pure ReO3.

UV-Vis absorption spectra (taken using V-670 spectrometer, Jasco Corp., JAPAN) of Re NPs are given in Figure 4(a) and 4(b). It has been observed that, in the Re / ReO_3_ NPs mixture, absorption spectra of Re NPs is dominated by UV-Vis spectra. The obtained absorption spectrum of the ReO_3_ NPs produced in pure water is shown in Figure 4(b), which presents an absorption peak around 510 nm. It has been observed that the obtained absorption peak is compatible with the literature [26].

Band gap values for Re / ReO_3_ NPs produced in this work were calculated from the absorption data by using Tauc-plot (hν–(αhν)2) given in Figure 4 [38–40] to be 4.71 eV and 4.36 eV for Re/ReO_3_ mixture and pure ReO_3_, respectively. It can be concluded that changes in the Tauc-plot indicate that the Re NPs dominates the UV-Vis spectrum. The band gap energy values obtained are also compatible with the literature [34,41,42]. It was found that the absorption spectra of Re/ReO_3_ mixture and pure ReO_3_ NPs were varied within the range from ultraviolet region to infrared region in spectrum depending on the synthesis parameters.

## 4. Conclusion

In recent years, rhenium NPs have been used for tumor treating therapies and coatings (plastics, metals, textiles) technologies. In addition, magnetic rhenium NPs have been used as a contrast agent and was produced using both methods of pulsed-laser decomposition of ammonium perrhenate or gamma radiation of dirhenium decacarbonyl. 

As a summary, in this study, it has been reported that the Re / ReO_3_ NPs mixture or ReO_3_ NPs were produced for the first time in the literature using the fsPLAL method. The produced NPs were characterized by XRD, SEM, TEM, EDX, and UV-VIS absorption spectroscopy methods. Re / ReO_3_ NP mixtures can also be produced changing laser irradiation time and reducing the liquid volume. Pure ReO_3_ NPs can be produced in the high laser fluence regime. XRD results show that pure ReO_3_ NPs have perovskite-like cubic structure. Produced particles have a spherical shape and particle sizes change from ~ 20 nm to ~ 60 nm due to the TEM results. SEM results present that micron-sized particles can be aggregated due to the evaporation of the liquid by heating when a large volume of water is used. Wide band gap materials are used in very broad application areas in semiconductor devices, optoelectronic devices, photodetectors, catalytic applications, chemical sensors etc. ReO_3_ NPs show a broad absorption band (from 350 nm up to 850 nm), and it can be emphasized that this may indeed be useful for sensors, diodes, and solar cell as photonic applications.
